# Nebulized glycyrrhizin/enoxolone drug modulates IL-17A in COVID-19 patients: a randomized clinical trial

**DOI:** 10.3389/fimmu.2023.1282280

**Published:** 2024-01-12

**Authors:** Ulises Zendejas-Hernandez, Nemi Alcántara-Martínez, Diana Tovar Vivar, Fermín Valenzuela, Alejandro Sosa Espinoza, Eduardo Emir Cervera Ceballos

**Affiliations:** ^1^ Research Department, SPV TIMSER, S.A.P.I. de C.V., Mexico City, Mexico; ^2^ Science Faculty, National Autonomous University of Mexico, Mexico City, Mexico; ^3^ Research and Development Department, Columbia Laboratories, Mexico City, Mexico; ^4^ National Cancerology Institute, Teaching department, Mexico, Mexico

**Keywords:** glycyrrhizin, enoxolone, immunomodulation, SARS-CoV-2 infection, IL-17A interleukin, inflammatory cytokines, COVID-19, IFN-gamma

## Abstract

**Introduction:**

Glycyrrhizin (GA) and its derivative Enoxolone (18β), isolated from the *Glycyrrhiza glabra* plant, are two potential molecules for treating viral diseases. Both demonstrate to regulate immune system with antiviral and anti-inflammatory activities, with the latter mainly due to modulation of inflammatory cytokines. The aim of this clinical trial was to evaluate the safety and efficacy of a nebulized GA/18β drug for treating COVID-19 patients.

**Methods:**

An open label, randomized, placebo-controlled clinical trial was conducted in Mexico City from January-August 2022 (Registration No. PROTAP-CLI-00). Clinical and biochemical parameters were recorded. Blood samples from patients were regularly collected to evaluate interleukins IL-4, IL-2, IL-1b, TNF-α, IL-17A, IL-6, IL-10,IFN-γ, IL-12, IL-8 and TGF-β1, as well as IgM and IgG against SARS-CoV-2. Two doses of the drug were used - 30/2 mg (dose A) and 90/4 mg (dose B).

**Results and discussion:**

Both GA/18β doses modulated inflammatory response by reducing mainly IL-17A expression, which in turn kept IL-1β, IL-6, IL-8 and TNF-α interleukins unchanged, indicating significant modulation of key interleukin levels to prevent exacerbation of the immune response in COVID-19 patients. Early on, dose A increased IgM, while dose B induced expression of the antiviral IFN-γ. No severe side effects were seen with either dose, indicating nebulized GA/18β is a safe treatment that could be used for COVID-19 and potentially other viral infections involving inflammatory response.

## 1 Introduction

Since the initial detection of severe acute respiratory syndrome coronavirus 2 (SARS-CoV-2) in December 2019 in Wuhan, China, the rapid transmission of novel coronavirus disease 2019 (COVID-19) has prompted investigations into various drugs for treating this viral respiratory infection. Despite extensive efforts, a safe and effective treatment for COVID-19 remains elusive. This underscores the need for alternative therapeutic agents targeting SARS-CoV-2 infection. Two promising candidates originating from the *Glycyrrhiza glabra* Linn. plant are Glycyrrhizinic acid (GA; CAS number 1405-86-3) and its derivative Glycyrrhetinic acid, also known as enoxolone (18β; CAS number 471-53-4). These molecules have displayed diverse therapeutic potential in SARS-infected cells and animal models (1).

Notably, both GA and 18β exhibit antiviral properties against SARS-CoV-2, employing distinct mechanisms to hinder viral entry and replication within cells (2, 3). For instance, these compounds can bind to viral proteins. Binding to the SARS-CoV-2 spike protein inhibits the interaction between the receptor-binding domain (RBD) and its receptor, angiotensin-converting enzyme 2 (ACE2), consequently preventing the virus from entering the cell. Meanwhile, binding to the SARS-CoV-2 main protease (Mpro) inhibits viral replication and assembly. GA and 18β can also bind to cell proteins, such as the ACE2 receptor and the type II transmembrane serine protease (TMPRSS2) (**Supplementary Figure S1**). This enzyme is not only a key player in virus entry for coronaviruses but also for influenza viruses (4–6).

GA and 18β exhibit anti-inflammatory and immune response effects through the modulation of cytokines, including interferon-γ (IFN-γ), tumor necrosis factor-α (TNF-α), interleukins (IL) such as IL-17, IL-1β, IL-4, IL-5, IL-6, IL-8, IL-10, and IL-12. Additionally, they affect intercellular adhesion molecules and transcription factors. This cytokine-mediated modulation may occur through different mechanisms, such as reducing the activity of the inflammatory mediator toll-like receptor (TLR), preventing virus binding to the ACE2 receptor (7), and inhibiting several high mobility group box 1 (HMGB1)-mediated pathological pathways by directly binding to the HMGB1 protein (**Supplementary Figure S1**) (2, 8).

Of particular interest, IL-17, a pro-inflammatory cytokine, has been observed at elevated levels in the peripheral blood of SARS-CoV-2 infected patients. This cytokine can trigger various inflammatory cytokines, including IL-6, IL-1β, IL-1, and IL-8 (9, 10). Encouragingly, insights from multiple clinical trials suggest that inhibiting IL-17 could hold therapeutic promise for inflammatory conditions (11).

The anti-inflammatory activity of GA and 18β has also been demonstrated in the context of respiratory infections caused by other viruses, such as influenza and respiratory syncytial virus (12, 13). The therapeutic activities of both GA and 18β suggests their viability as effective agents for treating respiratory viral infections like COVID-19. This potential stems from their dual immunomodulatory effect during the two phases of immune response observed in the course of COVID-19 disease. The initial phase, occurring during viral incubation, involves a specific adaptive immune response essential for virus elimination and the prevention of disease progression to severe stages. The subsequent phase is marked by extensive inflammation resulting from a compromised immune response, leading to elevated inflammatory cytokines and culminating in a cytokine storm, tissue damage, multi-organ failure, and eventual immune exhaustion (14, 15).

Both GA and 18β possess therapeutic and pharmacological attributes, including non-cytotoxicity, non-carcinogenicity, and a safe dosage range (3). As such, they have been successfully employed in combination with other compounds for the treatment of various conditions, such as erythrodermic psoriasis, hand hyperpigmentation (16), and chronic viral hepatitis in humans (17). While the potent antiviral and anti-inflammatory properties of GA and 18β in cellular and animal models are well-documented (18), their utilization for human respiratory tract infections remains limited.

Notably, both compounds are components of herbal formulations containing GA and 18β (as *Glycyrrhiza* sp. extract), found in Traditional Chinese Medicines for treating SARS-CoV-2 infection (19–21). These formulation, however, comprises additional medicinal plants aside from *Glycyrrhiza* sp. extract, making it unclear how GA and 18β individually impact inflammation process. Limited reports exist concerning the application of GA and 18β to treat SARS-CoV-2 infection in humans. In China, Diammonium Glycyrrhizinate, the diammonium salt of GA, was orally administered alongside a corticosteroid to a COVID-19 patient, resulting in alleviation of severe symptoms within 12 hours (22). Similarly, a European clinical trial demonstrated curative effects of a vaporizer solution containing 18β and GA, leading to symptom relief within 48 hours in COVID-19 positive subjects with severe symptoms (23). However, these studies lack assessments of treatment effects on inflammation molecules like interleukins, essential for comprehending the principal therapeutic mechanisms of GA and 18β in COVID-19 patients.

Furthermore, the mechanism of action of GA and 18β against the SARS-CoV-2 virus in humans remains inadequately understood, and their administration via nebulization has not been extensively explored. Inhalation-based drug delivery presents a promising avenue to address a key challenge associated with hydrophobic molecules like 18β, enhancing solubility and permeability for improved bioavailability while achieving high local concentrations in the respiratory system to combat viral infection without inducing side effects (24). Previous preclinical *in-vitro* studies conducted by us demonstrated that GA and 18β are safe and have a strong capability to inhibit the Spike-ACE2 protein binding. *In-vivo* experiments in a murine model showed that low and high nebulized GA/18β doses do not produce irritation or damage in the upper and lower airways (24). Therefore, a phase I trial, authorized by the Mexican regulatory agency (COFEPRIS: Federal Commission for the Protection against Sanitary Risks), investigated the effects of a nebulized drug containing GA and 18β molecules (GA/18β) on healthy subjects, revealing no adverse effects during administration and follow-up in humans (data not shown). Based on these preclinical and clinical trials, we proceeded to assess the tolerability, safety, and efficacy of this nebulized GA/18β drug in treating COVID-19 patients. This study aims to evaluate the efficacy of the GA/18β treatment by analyzing the immune system’s response, including the regulation of cytokines and SARS-CoV-2 antibodies throughout the disease progression.

## 2 Materials and methods

### 2.1 Study design

This phase 2 study was a randomized, placebo-controlled open clinical trial conducted in México City from January to August 2022 by SPV TIMSER company, when the main circulating SARS-CoV-2 variant in México was Omicron (25). The trial’s registration with COFEPRIS is documented as Registration No. PROTAP-CLI-00. The trial adhered to ethical principles in accordance with the Declaration of Helsinki and Good Clinical Practice guidelines.

Using G*Power software version 3.1.9.7, a minimum sample size of 20 patients per group was determined with an α of 0.05. Sixty-five COVID-19 positive subjects were recruited and randomized into the following 3 treatment groups: Group A received dose A of 30/2 mg GA/18β (n=20); Group B received dose B of 90/4 mg GA/18β (n=22); and the control group received 0.9% saline solution (n=23).

**Figure 1:**
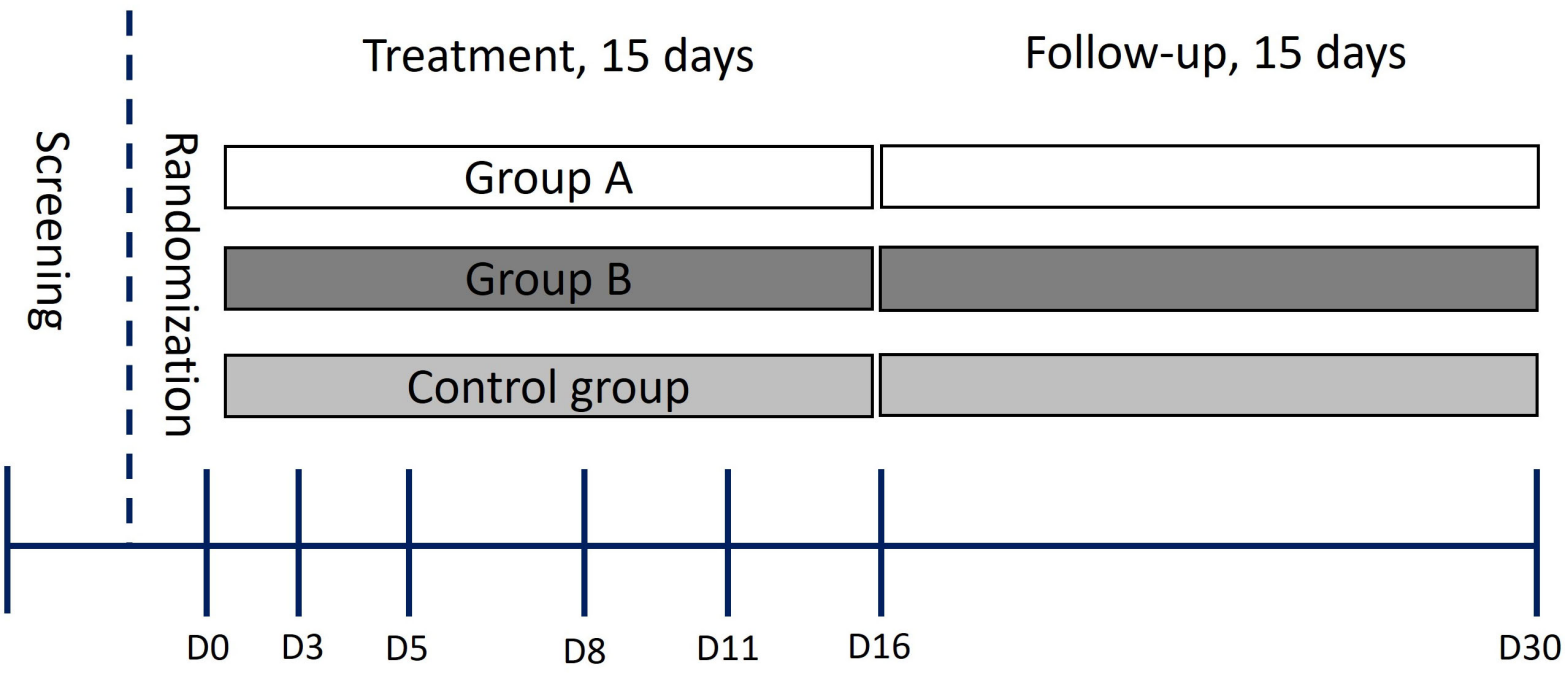
Schematic of the trial design. D0-D30 indicates sample collection days.

COVID‐19 positive status was confirmed through PCR testing, with enrolled patients exhibiting mild-moderate disease severity in accordance with the World Health Organization’s Clinical Guide for COVID-19 treatment (2021) (26). During a screening visit three days prior to treatment initiation, subjects meeting key inclusion criteria were assigned in a 1:1:1 ratio to the three groups. Treatment lasted for 15 consecutive days (**Figure 1**). Exclusions were made for patients failing inclusion criteria, while discontinuations occurred due to work-related reasons (**Supplementary Figure S2**). At the conclusion of the treatment phase, subjects were evaluated again for an additional 15 days (**Figure 1**). Throughout the treatment and follow-up periods, parameters related to safety, tolerability, side effects, and immune response were assessed.

### 2.2 Patients and intervention method

Eligible participants encompassed both women and men aged over 18, with a body mass index between 18 kg/m^2^ and 40 kg/m^2^. All patients provided informed consent and demonstrated willingness and capacity to adhere to study procedures and follow-up requirements. All subjects were confirmed with a diagnosis of mild-moderate COVID-19 and fulfilled the comprehensive eligibility criteria outlined in **Table 1**. Baseline demographic information, vital signs, and blood chemistry measurements were acquired for each group on day 0, prior to commencing treatment (**Supplementary Table S1**).

**Table 1:** Inclusion and exclusion criteria for patients.

Inclusion criteria	
• Ability to understand and provide informed consent
• Men and women aged 18 years or older, with body mass index between 18-40 kg/m^2^
• Confirmed diagnosis of COVID-19
• Positive PCR and/or antigen test for SARS-CoV-2 detection within 72 hours of screening visit
• Presenting with mild respiratory symptoms requiring medical management at screening • Willingness and ability to comply with all the procedures and follow-up of the study.
Exclusion criteria	
• Uncontrolled medical conditions • Electrocardiogram indicating potentially dangerous cardiac conditions that could endanger the patient during the study • Any condition, in the judgment of the principal investigator, that could compromise patient safety, interfere with treatment evaluation, or impact study results • Prior treatment with or consumption of any substances containing GA or 18β within 1 month before screening • Medical treatment including hormonal contraceptives, antivirals, and corticosteroids, except nutritional supplements and medications for chronic condition control

The administration of treatment occurred via nebulization utilizing a NEBUCOR compressor (P-103 model). Patients inhaled a solution consisting of 1 mL of the drug mixed with 4 mL of 0.9% saline solution (groups A and B), or 5ml of 0.9% saline solution as specified (control group), for a duration of 18-20 minutes, repeated every 24 hours over a span of 15 consecutive days.

### 2.3 Laboratory analyses

#### 2.3.1 Safety and tolerability measures

To assess the adverse effects, safety, and tolerability of the GA/18β drug, we examined biochemical markers related to liver and kidney function, fluid and electrolyte balance, glucose levels, and vital signs. Blood samples were collected using 5.6 mL and 4 mL Vacutainer tubes and stored at -70°C until analysis of parameters at certified clinical laboratories in Mexico City.

Vital sign parameters were quantified at days 0-16 and day 30, while laboratory health markers were measured at days 0, 3, 8, 16, and 30 to identify potential changes throughout the treatment and follow-up period.

Adverse events (AEs) included electrolyte imbalances or shifts in blood chemistry parameters, as well as both serious and non-serious events in accordance with the CTCAE v 5.0 Common Terminology Criteria for Adverse Events scale. AEs were documented through exploration, directed questioning, and assessment of laboratory analyses. Clinical evaluation of AEs, utilization, and discontinuation of concomitant medications were recorded from the initial day of treatment through the final day of the follow-up period.

#### 2.3.2 Quantification of interleukins and antibodies

The assessment of the GA/18β drug’s efficacy against SARS-CoV-2 infection involved the examination of its influence on inflammatory and immune responses. Blood samples underwent centrifugation at 4°C and 4500 r.p.m. for 5 minutes to isolate plasma, which was promptly stored at -70°C ± 10°C until processing for the quantification of cytokines and antibodies against SARS‐CoV‐2 (IgM and IgG) at 0, 3, 5, 8, 11, 16 and 30 days.

Serum levels of cytokines, namely IL-4, IL-2, IL-1β, TNF-α, IL-17A, IL-6, IL-10, IFN-γ, IL-12, IL-8, and TGF-β1, were quantified utilizing the Immunoassay Legendplex (Biolegend) Kit, adhering to the manufacturer’s guidelines. The concentration of interleukins was determined by establishing a standard curve with an R value > 0.95. Antibodies were detected using an ELISA test, following the method described by Stadlbauer et al. (27).

### 2.4 Statistical analysis

Categorical variables, including adverse events (AEs), utilization, and discontinuation of concomitant medicine, were summarized as percentages within their respective categories. Biochemical parameters were subjected to multiple analysis. For the evaluation of interleukin modulation during disease progression, the relative amount of each interleukin concerning its baseline value on day 0 was computed for each subject. This data normalization was conducted to assess the increase or decrease in the levels of each measured interleukin and identify possible significant differences between groups. An increase in interleukin levels was identified when the relative amount exceeded or equaled 140%, whereas a decrease was established when the relative amount was 50% or more. To identify significant differences among groups, a repeated-measures analysis of variance (ANOVA) was employed to analyze antibody titers and relative interleukin amounts. Subsequent pairwise comparisons of group means were performed using Tukey’s multiple range test. Statistical significance was attributed to values with p ≤ 0.05. To analyze the influence among the main measured interleukins, a Spearman correlation analysis was performed on the concentration data of IL-6, IL-8, IL-1β, and TNF-α in relation to IL-17A for each group. Correlation coefficients (r_s_) and significant correlations are indicated within the text (*p* < 0.0001***; *p* < 0.001**; *p* < 0.01*). The statistical analyses were conducted using GraphPad Prism 8.0 (GraphPad Software, Inc., CA).

## 3 Results

### 3.1 Safety and tolerability

Based on the percentage of subjects reporting AEs, the most frequent AEs during treatment and follow-up included headache, myalgia, dizziness, and paresthesia. Nevertheless, none of these AEs exceeded 8% within any group. Establishing definitive causality proved challenging due to the overlap of many symptoms with those of COVID-19, including headache, myalgia, dizziness, and paresthesia. For instance, myalgia was reported in both control group and group A subjects at a similar proportion (5.3% of the total patients). Consequently, AEs were attributed to the GA/18β-treated subjects and not the control group. These AEs encompassed dyspnea, scratchy throat, sialorrhea, nausea, tachycardia, diarrhea, and low back pain. Among these, dyspnea and itchy throat were the most frequent at 8.8%, while the rest of AEs were each reported by a single patient (1.8%). Importantly, all reported AEs were classified as non-serious and mild to moderate intensity. Furthermore, most of these AEs (sialorrhea, nausea, tachycardia, and diarrhea) were not reported beyond day 5.

### 3.2 Effect of GA/18β treatments

**Figure 2:**
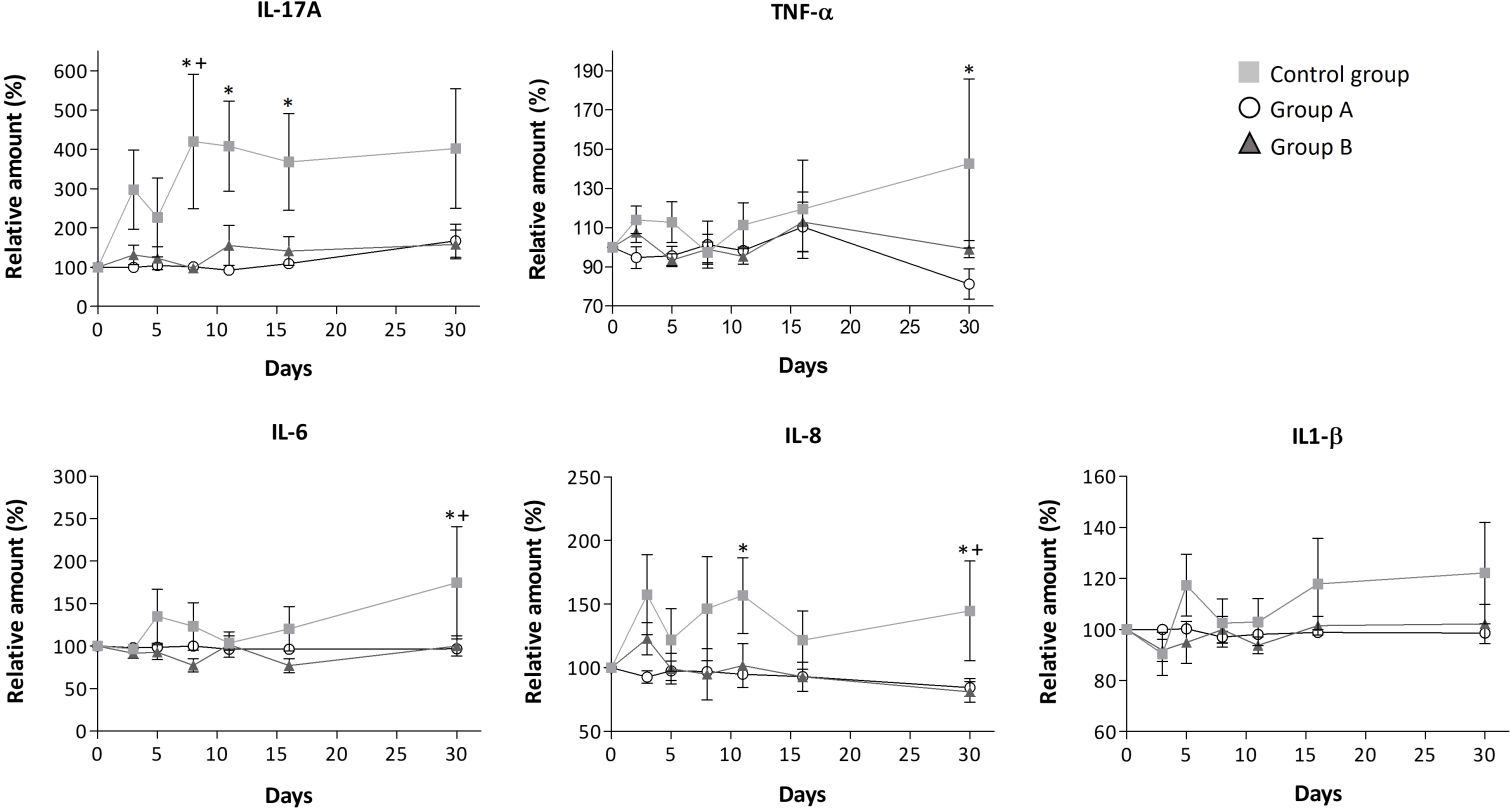
Modulation of serum levels of IL-17A, TNF-α, IL-6, IL-8, and IL-1βfor each group. Mean ± standard error values are reported. Statistically significant differences compared to control are indicated by *(group A) or + (group B) when p ≤ 0.05. The concentration data, the lower and upper values, and limits of detection for these interleukins are shown in **Supplementary Table S3**.

**Figure 3:**
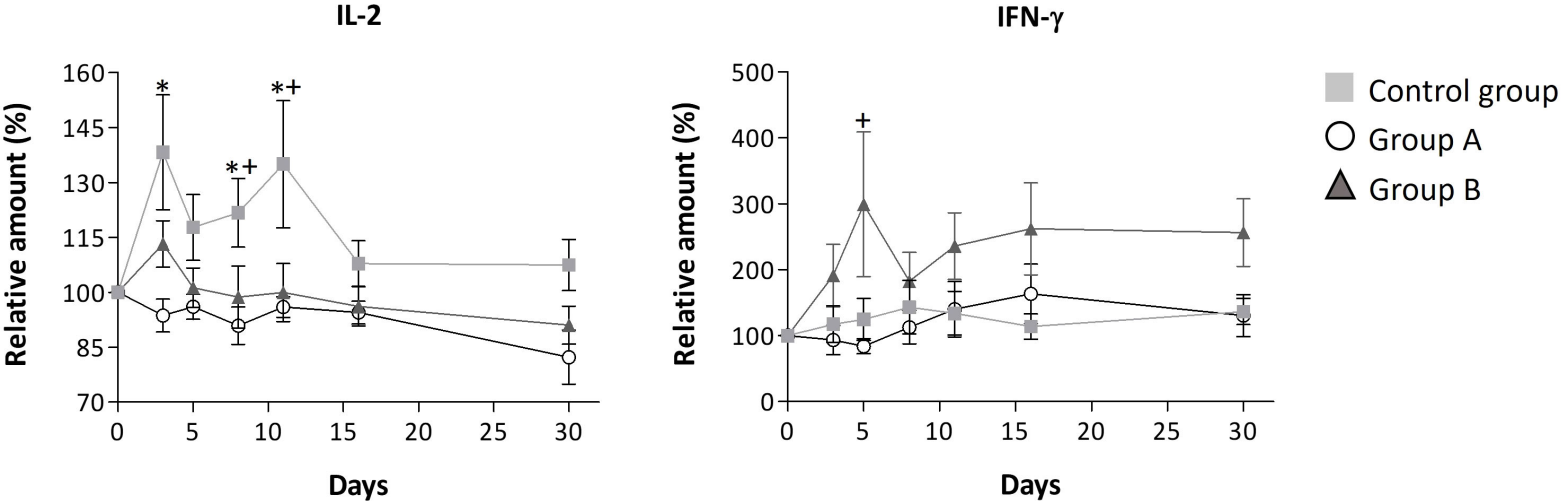
Relative serum levels of IL-2 and IFN-γ over disease progression. Mean ± standard error values are reported. Statistically significant differences compared to control are indicated by * (group A) or + (group B) when p ≤ 0.05. The concentration data, the lower and upper values, and limits of detection for these interleukins are shown in **Supplementary Table S3**.

Our findings highlight the immunomodulatory effects of the GA/18β drug, as evidenced through the assessment of interleukins and SARS-CoV-2 specific antibodies. In general, it has been reported that interleukins are molecules with high variation in basal levels due to various subject-specific conditions, such as sex, comorbidities, initial symptoms that could differ concerning the onset of the infection, sample collection time, and other factors. These variables can consequently increase the standard error of the data (28–31). However, the analysis of interleukins revealed that the treatments tended to reduce the increase in interleukin levels. Overall, this phenomenon was observed in the high percentage of the measured interleukins (82% and 55% in group A and B, respectively) which levels were decreased compared to the control group. A particularly significant decrease was observed in IL-17A, IL-2, IL-8, IL-6, and TNF-α interleukins for both groups, as depicted in **Figures 2**, **3**. Unlike the treated groups, the relative amount of IL-17A showed a significant increase from day 8 in the control group. The obtained Spearman’s coefficient demonstrates positive correlations of IL-17A with IL-8 (r_s_=0.574***), IL-6 (r_s_=0.274**), and TNF-α (r_s_=0.721***) for this group.

Furthermore, it was observed that the administration of GA/18β doses led to a reduction in the intake of concomitant medications. This suggests that the GA/18β drug mitigates symptoms in patients during the 15-day treatment period. Notably, within the initial 7 days, when COVID-19 symptoms are most pronounced, nebulized treatment contributed to a decrease in the number of patients requiring concomitant medications. During the first nebulization week, discontinuation of concomitant medication use was observed in 100%, 94%, and 77% of subjects receiving treatments doses A, B, and control group, respectively (**Table 2**).

**Table 2:** Use and discontinuation of concomitant medications during the trial by treatment group.

	Groups		
Use/discontinuation of concomitant medicine	Control	A	B
Use during 0-15 days	95.0	73.6	85.0
Discontinuation on examination day	0	35.7	17.7
Discontinuation at day 7	76.5	100.0	94.1
Discontinuation after day 7	23.5	0.0	5.9

Discontinuation percentages were calculated based on the total number of subjects using concomitant medicines. Values are shown as percentages.

### 3.3 Effect of dose A

**Figure 4:**
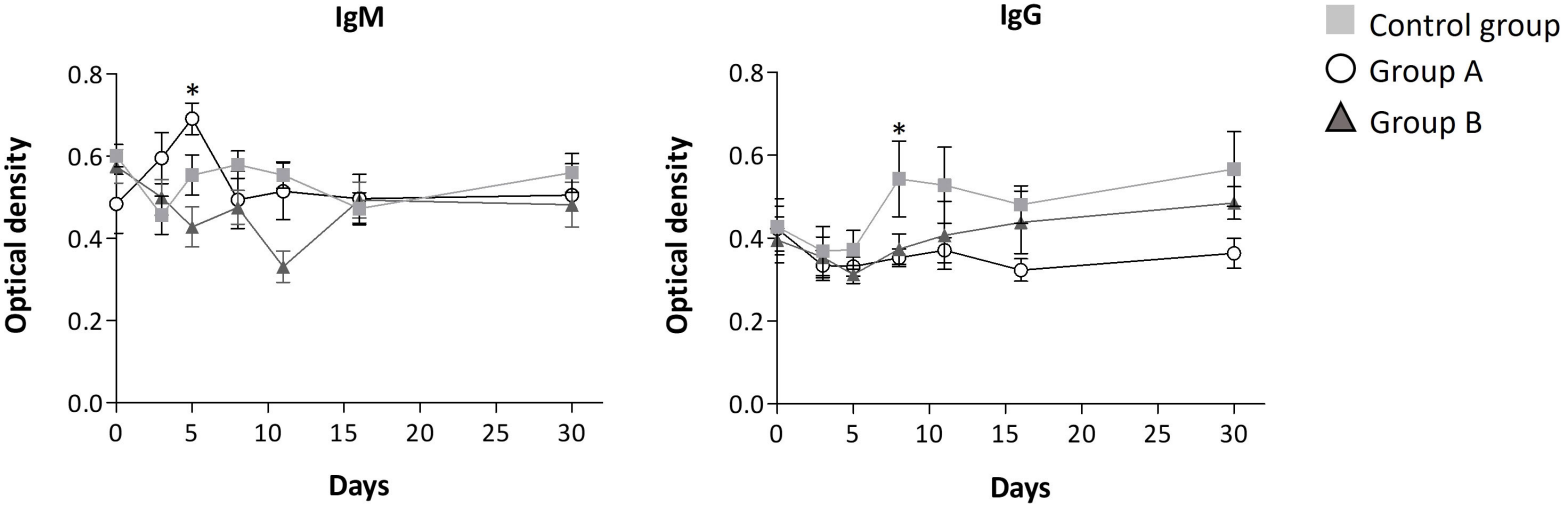
Antibody level progression. Mean ± standard error values are reported. Statistically significant differences compared to control are indicated by * (group A) when p ≤ 0.05.

Throughout the disease progression, in group A, most of the interleukins (IL-4, IL-2, IL-1β, IL-6, IL-8, IL-12, IL-10, IL-17A, TNF-α, and TGF-β1) remained unchanged, as indicated by **Figures 2**, **3**; **Table 3**. Dose A had a notable inhibitory effect on the production of specific interleukins, including IL-6, IL-17A, IL-8, IL-10, and IL-2, as their levels were significantly reduced compared to the control group (**Figures 2**, **3**; **Table 3**). Among these, IL-17A experienced the most pronounced reduction, with its levels decreasing by up to 4.4 times, maintaining a consistent relative value of approximately 100% throughout the disease course (**Figure 2**). The recorded Spearman correlation coefficients between IL-17A and TNF-α (r_s_ = 0.385***) and between IL-17A and IL-6 (r_s_ = 0.344***) suggest a positive relation between these interleukins (**Supplementary Table S4**). Moreover, dose A demonstrated an influence on antibody levels. The IgG concentration remained similar to that of the control group until day 5, after which a subsequent significant decline of approximately 1.4-1.6 fold compared to the control group was observed, reaching around 100%. This suggests a lack of temporal significant differences. In contrast to what was observed in IgG, treatment with dose A led to an early and significant surge in IgM levels by day 5 (**Figure 4**), reaching a relative value of 200%.

**Table 3:** Modulation of relative IL-4, IL-12, TGF-β1, and IL-10 cytokines levels over time.

Cytokines	Days after starting treatment						
	3	5	8	11	16	30	Group
IL-4	105 ± 10.4	100 ± 12.2	102 ± 8.7	118 ± 16.1	95 ± 11.7	116 ± 15	Control
	100 ± 0.4	95 ± 4.8	96 ± 3.5	93 ± 5.4	97 ± 2.0	100 ± 0.37	A
	130 ± 29	118 ± 19.9	126 ± 28.5	116 ± 12.5	135 ± 29.9	122 ± 12.2	B
IL-10	106 ± 8.9	103 ± 8.3	108 ± 10.0	116 ± 13.2	114 ± 12.3	125 ± 12.6	Control
	96 ± 5.5	110 ± 12.8	89 ± 4.4	97 ± 4.0	103 ± 5.0	96 ± 9.4*	A
	108 ± 9.3	102 ± 11.8	103 ± 9.1	114 ± 13.2	116 ± 13.2	127 ± 11.8	B
IL-12	106 ± 8.6	105 ± 9.5	117 ± 11.7	124 ± 13.1	121 ± 13.4	133 ± 13.1	Control
	101 ± 1.5	102 ± 1.5	92 ± 5.3	92 ± 4.8	100 ± 7.6	130 ± 24.6	A
	132 ± 24.9	136 ± 25.9	110 ± 11.3	147 ± 27	134 ± 14.8	140 ± 12.5	B
TGF-β1	104 ± 14.1	130 ± 18.2	116 ± 20.3	121 ± 16.0	131 ± 16.5	134 ± 18.0	Control
	106 ± 3.9	97 ± 2.4	94 ± 6.2	107 ± 15.3	92 ± 5.2	93 ± 5.1	A
	84 ± 6.1	109 ± 11.6	143 ± 30.1	148 ± 25.6	115 ± 17.6	100 ± 13.2	B

Relative amounts were calculated as a percentage of the baseline value on day 0. Mean ± standard error values are reported for each interleukin. Statistically significant differences compared to the control group are indicated by an asterisk (*) when p ≤ 0.05.

### 3.4 Effect of dose B

The higher dosage led to an elevation in the relative amount of three interleukins (IL-4, IL-12, and IFN-γ) when compared to the control group, as depicted in **Figure 3** and **Table 3**. However, among these, only IFN-γ exhibited a significant modulation, demonstrating a remarkable increase to 300% of its relative amount (**Figure 3**). In contrast, a level reduction amounting to 55% of the total interleukins was noted when compared to the control group. This modulation was particularly significant for IL-17A, IL-6, IL-8, and IL-2 (**Figures 2**, **3**). In parallel with observations from dose A, IL-17A experienced the most substantial reduction among the interleukins, demonstrating a decrease of up to 4.3 times with dose B when compared to the control group. Remarkably, IL-17A’s relative amount remained relatively stable at around 100% throughout the disease progression (**Figure 2**). The recorded Spearman correlation coefficients of IL-17A with TNF-α (r_s_ = 0.798***), IL-6 (r_s_ = 0.366***), and IL-8 (r_s_ = 0.277**) suggest positive correlations of IL-17A with these interleukins, while the negative coefficient value for IL-17A with IL-1β (r_s_ = -0.387***) suggests a negative correlation between these two cytokines (**Supplementary Table S4**).Treatment with dose B also elicited modifications in antibodies. The concentration of IgM closely resembled that observed in the control group and exhibited negligible changes over time. While IgG values exhibited a tendency to rise from day 11 to day 30, they consistently remained 1-1.4 times lower than those found in the control group (**Figure 4**).

## 4 Discussion

### 4.1 Safety of Nebulized GA/18β treatment

One of the aims of this study was to evaluate the safety and tolerability of GA/18β treatments in patients with COVID-19. Although some side effects were observed in patients treated with the GA/18β drug, the majority of these symptoms were common across both the GA/18β treated groups and the control group. These symptoms were likely attributed to the underlying COVID-19 symptomatology. Conversely, the infrequent occurrence and mild intensity of AEs in the GA/18β treated groups, in contrast to the absence of such AEs in the control group, coupled with the lack of significant changes in biochemical parameters (**Supplementary Table S2**), indicate that the administered doses of GA/18β did not induce severe side effects.

These results can be linked to the chosen doses and administration method. Specifically, this trial utilized GA doses below 100 mg/day, a threshold below which GA metabolism is unlikely to yield severe side effects. Existing literature supports the safety of long-term dosages of up to 100 mg/day of GA in humans, devoid of the severe side effects associated with higher doses, such as hypermineralocorticoid-like effects (32, 33).

On another note, both GA and 18β, when dosed at levels sufficient to induce therapeutic effects, have demonstrated the propensity to cause side effects (34). Conversely, low dosages of these compounds have been successfully employed in conjunction with other molecules to achieve desired therapeutic effects. For instance, in a trial targeting COVID-19 subjects, a formulation containing GA (12.49 μg/day), 18β (2.49 μg/day), Resveratrol (18.75 μg/day), and Liquorice (424.8 µg/day) was administered for five days without any reported side effects (23). Hence, identifying the appropriate doses of GA and 18β that elicit antiviral and anti-inflammatory effects while maintaining safety remains a challenge in their application as treatment.

To devise an effective drug based on GA and 18β, nebulization was employed as the delivery method. This approach not only enhances the bioavailability of the active compounds but also achieves high local concentrations in the respiratory system without provoking severe side effects. This positive outcome can be attributed to the reduction in GA metabolism to its derivative, 18β, within the gastrointestinal tract when administered via nebulization rather than orally. This decrease in metabolism mitigates the deleterious effects associated with the structural similarity between 18β and corticosteroids (34–36). Mild and infrequent side effects, such as dyspnea and an itchy throat, reported in this trial could likely be attributed to the nebulization therapy itself, as certain aerosols can induce reactive bronchospasm and increased airway resistance, especially in patients with pre-existing respiratory conditions like COVID-19 (37).

Therefore, nebulized GA/18β not only achieves a high local concentration of these compounds, sufficient for a therapeutic effect in treating SARS-CoV-2 infection, but also prevents severe AEs. This underscores the safety of the GA/18β drug for the treatment of COVID-19 patients and potentially other viral infections as well.

### 4.2 GA/18β as an immunomodulator treatment

The nebulization of the GA/18β drug for the treatment of SARS-CoV-2 infection resulted in the modulation of cytokines that play a role in the progression of the illness, acting as either pro-inflammatory or antiviral inducers. Previous studies have demonstrated that both GA and 18β have the potential to control cytokine induction in inflammatory processes (5). This effect could be attributed to their chemical structure, which bears resemblance to corticosteroids, affording both molecules glucocorticoid-like properties, including immune modulation and suppression of inflammation (22). The suppression of the inflammatory response could probably be attributed to GA and 18β binding to the extracellular HMGB1 protein, which is released due to the activity of viral proteins of SARS-CoV-2 (5, 38, 39).

In addition to the modulation of interleukins, we observed alterations in the levels of SARS-CoV-2 antibodies due to the treatments. In COVID-19 patients, the lower dose of GA/18β increased IgM levels and maintained IgG levels with no significant fluctuations over time. This elevation in IgM levels might be connected to the aforementioned interleukin modulation, given that IgM is the initial immunoglobulin to emerge in the immune response, aiding in the clearance of pathogens during their early stages. This could potentially lead to a reduced inflammatory response in subsequent stages of COVID-19 progression (40).

Discrepancies in IgG levels between the two GA/18β treatment doses were also observed. The higher dose exhibited a tendency to elevate IgG levels starting from day 11, in comparison to the lower dose. Although this pattern was not significant, it was similar to the reported by Li and Zhou (41) in a mouse model of allergic rhinitis, where a higher GA dose led to increased IgG levels compared to a lower GA dose (41, 42). The modulation of inflammatory cytokines and antibodies by both doses of GA/18β likely contributes to the improvement in patient health, as evidenced by a majority of subjects discontinuing concomitant medication for COVID-19 symptoms within the initial 7 days of illness.

### 4.3 The administration of GA/18β treatments prevents the elevation of IL-17A and IL-2 interleukins

The modulation of cytokines, particularly IL-17A and IL-2, was observed in response to GA/18β treatments for SARS-CoV-2 infection. Emerging evidence underscores the vital role of IL-17 in COVID-19 pathogenesis, with its capacity to activate various pro-inflammatory cytokines like IL-6, IL-1β, IL-1, and IL-8 (9, 10). Notably, higher levels of IL-17 and Th17 cells (IL-17-producing cells) have been identified in severely hospitalized COVID-19 patients (43) and those with persistent symptoms (44). To date, reports of IL-17A levels in COVID-19 patients with mild/moderate infection are scarce. A recent study demonstrated that IL-17 is also increased in this severity of illness (45). Additionally, IL-17A upregulation has been implicated in the exacerbation of inflammatory conditions caused by other viruses such as influenza, respiratory syncytial virus, coxsackievirus, and coronaviruses, which subsequently enhances viral replication (46).

In our study, we observed a substantial induction of IL-17A in the control group of COVID-19 patients up to day 8, after which the IL-17A concentration remained significantly elevated compared to the GA/18β treated groups (**Figure 2**). This phenomenon could be attributed to the reported adaptive immunity of COVID-19 patients, whereby their neutrophils promote T-cell differentiation into Th17 cells (11).

Due to the known capacity of IL-17A to activate various cytokines and the positive correlation found between IL-17A and IL-6, IL-8, and TNF-α, the increase in IL-17A could potentially trigger dysregulated pro-inflammatory responses. This, in turn, may lead to elevated levels of the inflammatory cytokines IL-6, IL-8, and TNF-α in patients in the control group. Considering the reported role of TNF-α in stimulating inflammatory cytokines, it is suggested that the observed increase in TNF-α over time in non-GA/18β treated patients could also contribute to the significant increase in IL-6 and IL-8 release. While the observed increase in IL-1β in the control group was not statistically significant, it is probable that TNF-α and IL-17A could influence the increase in this cytokine. For example, in fibroblast cells, the interaction between IL-17 and low proportions of TNF-α has been observed to result in a synergistic pro-inflammatory effect, stimulating high concentrations of IL-6, IL-8, and IL-1β (47). Furthermore, the increased IL-6 production in the control group could enhance the generation of IL-17-producing Th17 cells (48), as evidence supports a linkage between IL-17 and IL-6-mediated activity in viral infections (10, 49). Although correlations between interleukins are shown in this study, more in-depth studies are necessary for a full understanding of the influence and interaction of IL-17A with other inflammatory cytokines in COVID-19 patients.

Conversely, in the GA/18β treated groups, we observed the attenuation of IL-6, IL-8, and IL-1β, likely resulting from the lower stimulation of IL-17A and TNF-α. This, in turn, could mitigate further pro-inflammatory stages (50). The inhibition of IL-17 could potentially expedite patient recovery, leading to reduced recovery time (51–54), which may explain the lower intake of concomitant medications in the initial days by the GA/18β treated groups. In fact, in different viral infection models like H1N1 influenza and respiratory syncytial virus, the knockout of IL-17 in murine models or the inhibition of IL-17 by monoclonal antibodies in humans has been linked to improved infection recovery and mitigation of related sequelae (51–53).

Reduction of IL-17 levels by dose B in SARS-CoV-2 infected subjects could probably result from two different pathways: inhibition of the HMGB1-mediated inflammation response, as GA and 18β can directly bind to HMGB1 (2, 8), and a possible inhibition of the viral infection in the early stages, attributed to the antiviral activity produced by early IFN-γ release (55). In various inflammation models, GA has been observed to diminish IL-17 concentrations. For instance, GA exhibits a protective role in hepatitis by reducing IL-17 production (56) and ameliorates colitis by attenuating IL-17-producing T-cell responses (39).

Another interleukin significantly influenced by the treatments was the proinflammatory cytokine IL-2, which participates in T-cell activation and the production of cytokines TNF-α and IFN-γ (57). Given that TNF-α triggers a range of cytokines and chemokines, the significant reduction in IL-2 levels in groups A and B suggests that GA/18β treatments could modulate the levels of inflammatory interleukins by reducing IL-2-mediated TNF-α. This observation aligns with findings from an animal model where different proportions of GA and 18β were found to inhibit IL-2 levels among other associated cytokines and chemokines (58).

Despite the overall reduction in IL-2 levels due to GA/18β treatments, it was observed that dose B induced IL-2, compared to dose A, in the initial 3 days of infection, a period when disease symptoms are most pronounced. Importantly, this IL-2 induction did not persist after day 3, possibly allowing for heightened IFN-γ production during the early stages of infection in the dose B-treated group (59).

### 4.4 GA/18β doses show different immunomodulation effects

While the modulation of IL-17A and IL-2 remains a shared outcome of treatments, important differences in immune system modulation across doses were observed. In particular, dose A exhibited a more robust immune system modulation compared to dose B, observed as non-significant increases in detected inflammatory interleukins over time. This modulation effect might be linked to IgM-mediated signaling pathways. The initial surge in IgM could potentially regulate interleukin concentrations, preventing cytokine imbalances (40).

Conversely, though IL-10 interleukin levels were comparable between dose A and B during drug administration, an early and modest IL-10 increase on day 5 followed by its subsequent significant reduction was detected in group A (**Table 3**). This could potentially function to curb excessive inflammation, as IL-10 has been reported to suppress the synthesis of proinflammatory cytokines in T-cells, activated macrophages, neutrophils, and monocytes (60–62).

While treatment A tended to maintain interleukin levels relatively close to baseline (100%) during the course of illness, the higher GA/18β dose induced an early increase in two cytokines with reported antiviral activity, namely IFN-γ and IL-12 interleukins. IFN-γ’s ability to hinder viral replication and exhibit anti-inflammatory properties suggests that its elevation induced by dose B might play a pivotal role in slowing down viral replication, limiting spread, and mitigating the early-stage inflammation response to SARS-CoV-2 infection (63). Notably, other studies in viral infections and asthma have also demonstrated IFN-γ modulation by GA or 18β (8, 64, 65).

The significant induction of IFN-γ in the initial days, as observed in group B, could potentially inhibit Th17 cell formation (55) and modulate IL-6, IL-8, and IL-12 concentrations (66). Elevated IFN-γ levels might decrease IL-6 and IL-8 (66) while increasing IL-12 levels, given the IFN-γ-IL-12 positive feedback loop reported to trigger IL-12 production (67, 68). Furthermore, in peritoneal macrophages, GA has been shown to stimulate IL-12 messenger RNA accumulation and IL-12 protein secretion during LPS-induced inflammation (67).

As IL-12 plays a vital role in the modulation of cytokines and T cell subsets (69), the observed early elevation of IL-12, while not statistically significant, may contribute to regulating the inflammation process in recipients of the higher dose.

The induction of IFN-γ in plasma and other interleukins with antiviral activity is not commonly observed in patients diagnosed with moderate-severe COVID-19; instead, they tend to decline in the early days of infection (63, 65). Thus, the GA/18β-mediated IFN-γ induction, alongside other demonstrated antiviral mechanisms of GA and 18β, such as the inhibition of key enzymes for virus replication and entry (Mpro and TMPRSS2 proteins, respectively), particularly during the initial days, could play a crucial role in reducing SARS-CoV-2 infection and associated symptoms.

## 5 Conclusion

In summary, our findings demonstrate that the two examined doses of nebulized GA/18β exhibit at least two mechanisms of action against mild-moderate COVID-19. First, it attenuates the inflammatory cascade, likely by reducing IL-17A which in turn modulates pro-inflammatory cytokine expression. Second, it demonstrates potential antiviral activity by promptly inducing IFN-γ. Importantly, the nebulized GA/18β formulation showed a favorable safety profile, affirming its suitability for treating COVID-19 and other viral infections characterized by inflammatory response. As far as we know, this is the first clinical trial investigating immune system modulation (interleukins and antibodies) in response to GA/18β treatment in COVID-19 patients. This study demonstrates the impact of nebulized GA/18β on key interleukins promoting inflammation. Further investigations in larger cohorts are needed to fully elucidate the therapeutic mechanisms of GA/18β treatment in COVID-19. Given the broad spectrum of biological activities GA and 18β exhibit against respiratory viral infections, these future inquiries hold potential for specific treatments advancement.

## Data availability statement

The original contributions presented in the study are included in the article/**Supplementary Material**. Further inquiries can be directed to the corresponding author.

## Ethics statement

The studies involving humans were approved by Investigation Committee of the Hospital Angeles del Pedregal and Research Ethics Committee of the Hospital Angeles del Pedregal. The studies were conducted in accordance with the local legislation and institutional requirements. The participants provided their written informed consent to participate in this study.

## Author contributions

UZ-H: Conceptualization, Methodology, Writing – review & editing, Formal analysis, Visualization, Writing – original draft. NA-M: Formal analysis, Visualization, Writing – original draft, Writing – review & editing. DT-V: Visualization, Writing – review & editing, Data curation, Investigation. FV: Visualization, Writing – review & editing, Methodology, Supervision. AS-E: Writing – review & editing, Data curation. EC: Writing – review & editing, Conceptualization, Methodology, Supervision.

## Supplementary material

Supplementary Figure 1 General scheme of antiviral and anti-inflammatory mechanisms of GA and 18β during SARS-CoV-2 infection. TLR, Inflammatory mediator toll-like receptor; ACE, Angiotensin-converting enzyme; ACE2, Angiotensin-converting enzyme 2; RAS, Renin–angiotensin system; TMPRSS2, Type II transmembrane serine protease; HMGB1, High mobility group box 1 protein; Mpro protein, SARS-CoV-2 main protease; ROS, Reactive oxygen species.
