# Supplementary figures and images for: Nebulized glycyrrhizin/enoxolone drug modulates IL-17A in COVID-19 patients: a randomized clinical trial
Source: Front Immunol. 2024 Jan 12;14:1282280. doi: 10.3389/fimmu.2023.1282280 (PMC10811189; doi:10.3389/fimmu.2023.1282280)

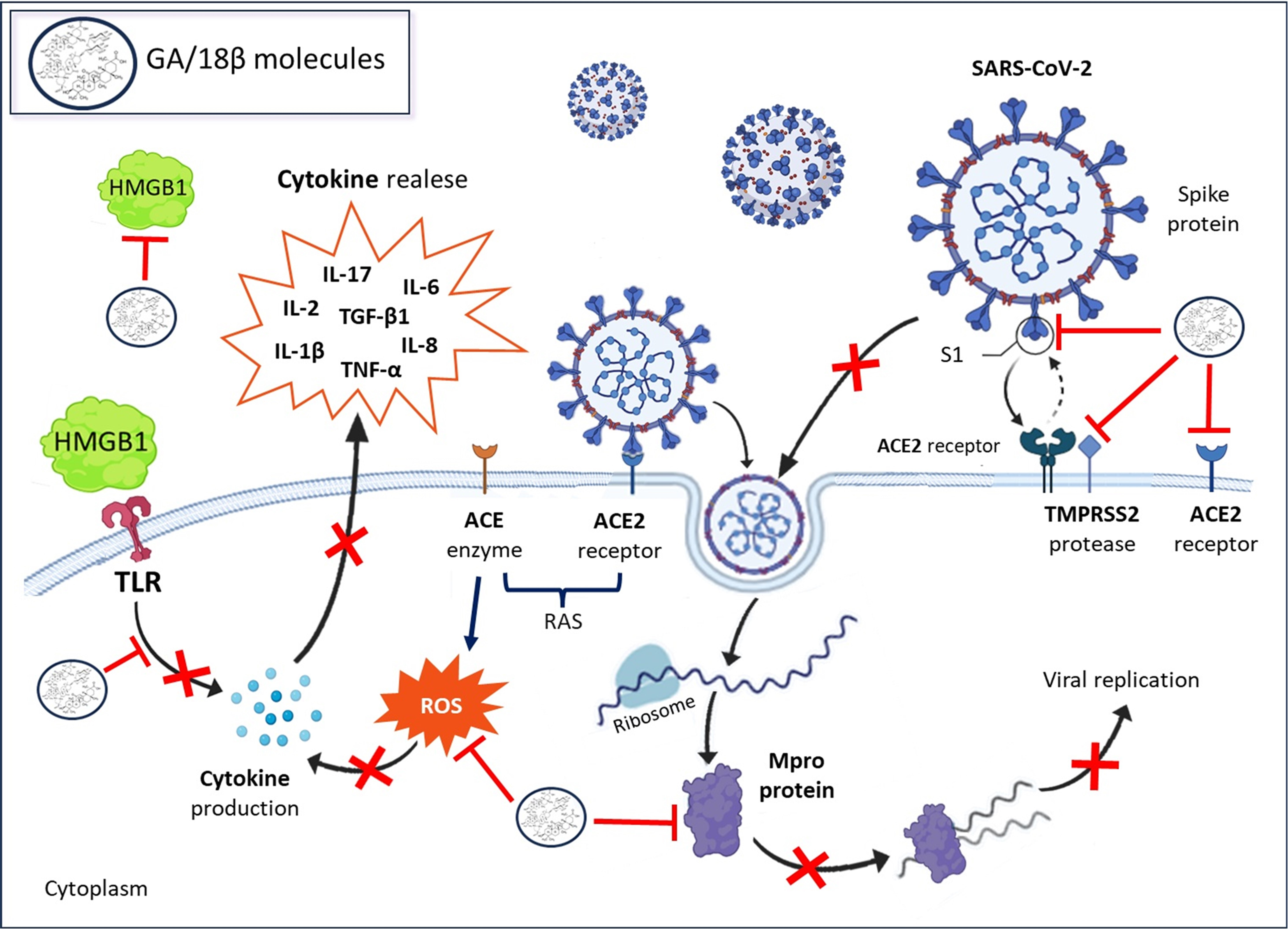

Supplement: Supplementary Figure 1 — General scheme of antiviral and anti-inflammatory mechanisms of GA and 18β during SARS-CoV-2 infection. TLR, Inflammatory mediator toll-like receptor; ACE, Angiotensin-converting enzyme; ACE2, Angiotensin-converting enzyme 2; RAS, Renin–angiotensin system; TMPRSS2, Type II transmembrane serine protease; HMGB1, High mobility group box 1 protein; Mpro protein, SARS-CoV-2 main protease; ROS, Reactive oxygen species. [file Image_1.tiff]
