# Supplementary material for: Nebulized glycyrrhizin/enoxolone drug modulates IL-17A in COVID-19 patients: a randomized clinical trial
Source: Front Immunol. 2024 Jan 12;14:1282280. doi: 10.3389/fimmu.2023.1282280 (PMC10811189; doi:10.3389/fimmu.2023.1282280)
Supplement: Supplementary file 2 [file Image_2.pdf]

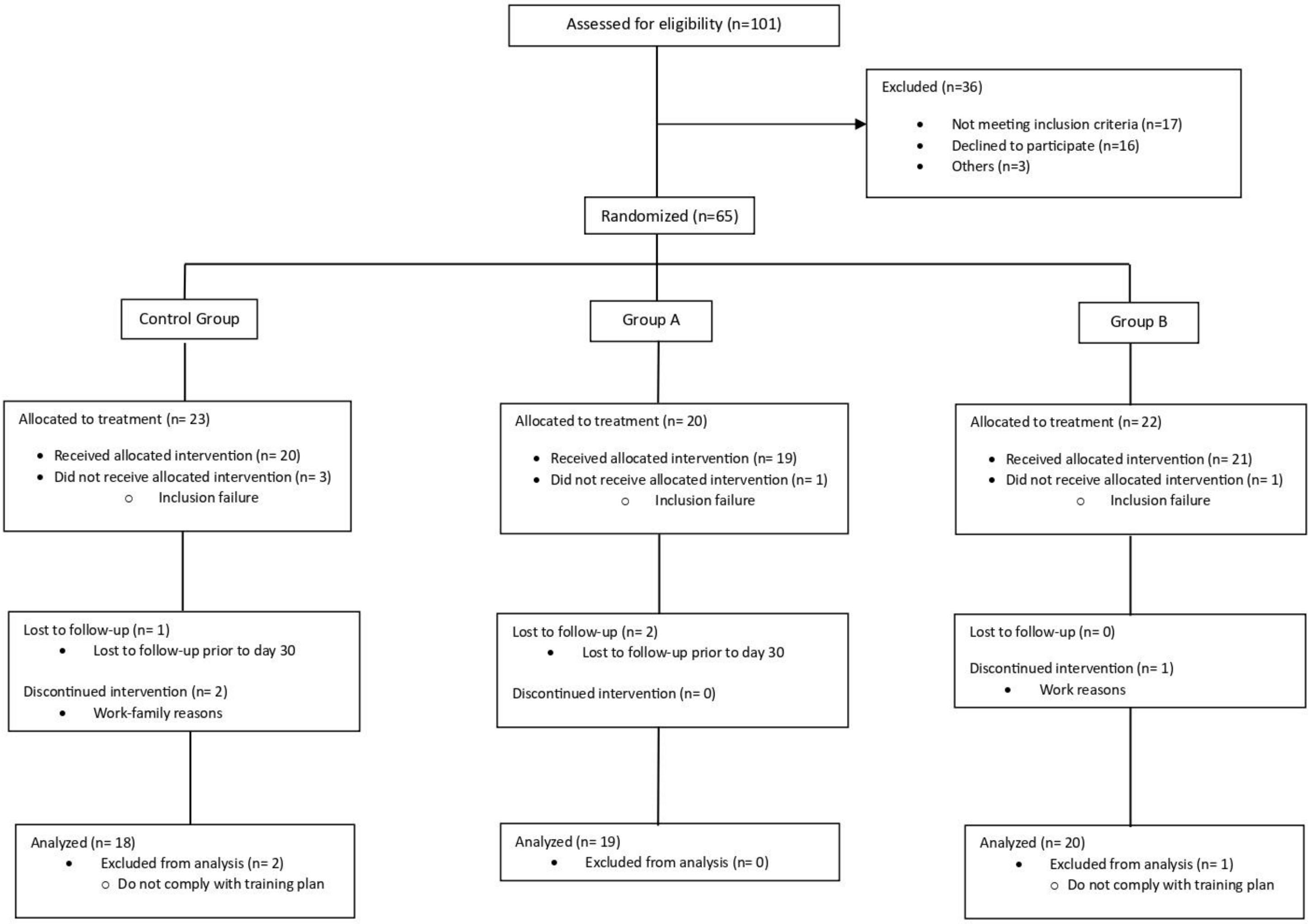

Supplementary Figure 2. Disposition of patients for each group. Control group (0.9% saline solution), Group A (30/2 mg GA/18β), Group B (90/4 mg GA/18β).
