# Supplementary material for: Nebulized glycyrrhizin/enoxolone drug modulates IL-17A in COVID-19 patients: a randomized clinical trial
Source: Front Immunol. 2024 Jan 12;14:1282280. doi: 10.3389/fimmu.2023.1282280 (PMC10811189; doi:10.3389/fimmu.2023.1282280)
Supplement: Supplementary file 3 [file Table_1.docx]

**Supplementary Table 1.** Baseline demographics, vital signs, and blood chemistry data of patients by group. The mean and standard deviation (SD) values are showed. Control group (0.9% saline solution), Group A (30/2 mg GA/18β), Group B (90/4 mg GA/18β).

| **Variable** | | **Group A** | **Group B** | **Control group** |
| --- | --- | --- | --- | --- |
| Sex, n (%) | Male | 6 (31.6) | 10 (50) | 10 (50) |
|  | Female | 13 (68.4) | 10 (50) | 10 (50) |
| Age (y) | | 38.6 (11.8) | 49.6 (18.6) | 40.5 (14.5) |
| Weight (kg) | | 68.1 (15.2) | 67.8 (9.8) | 73.4 (13.7) |
| Size (m) | | 1.61 (0.1) | 1.68 (0.1) | 1.65 (0.1) |
| BMI (kg/m^2^) | | 26.1 (4.5) | 24.0 (2.4) | 26.9 (4.2) |
| Systolic Blood Pressure (mm/Hg) | | 103 (9) | 107 (9) | 107 (9) |
| Diastolic Blood Pressure (mm/Hg) | | 68 (6) | 69 (7) | 69 (7) |
| Heart Rate (bpm) | | 74 (10) | 73 (14) | 76 (9) |
| Breathing Frequency (bpm) | | 18 (2) | 19 (1) | 19 (1) |
| Temperature (°C) | | 36.1 (0.7) | 36.4 (0.5) | 36.7 (0.5) |
| Oxygen saturation (%) | | 95 (2) | 94 (2) | 94 (2) |
| Glucose (mg/dl) | | 88.0 (17.29) | 86.15 (11.69) | 91.30 (10.97) |
| Glomerular Filtration Rate,  (mL/min/1.73 m^2^) | | 104.42 (17.67) | 95.91 (16.02) | 101.42 (17.06) |
| Urea (mg/dl) | | 23.79 (9.65) | 26.85 (7.91) | 24.45 (12.02) |
| Creatinine (mg/dl) | | 0.79 (0.23) | 0.83 (0.16) | 0.83 (0.12) |
| Uric Acid (mg/dl) | | 4.37 (1.15) | 4.73 (1.33) | 4.76 (1.75) |
| Cholesterol (mg/dl) | | 173.42 (26.36) | 168.30 (38.89) | 172.60 (34.82) |
| Triglycerides (mg/dl) | | 150.42 (57.05) | 99.80 (29.72) | 139.60 (56.00) |
| Total Bilirubin (mg/dl) | | 0.37 (0.15) | 0.49 (0.22) | 0.43 (0.31) |
| Direct Bilirubin (mg/dl) | | 0.15 (0.05) | 0.19 (0.06) | 0.17 (0.09) |
| Indirect Bilirubin (mg/dl) | | 0.23 (0.10) | 0.30 (0.16) | 0.26 (0.22) |
| Aspartate Aminotransferase (U/L) | | 31.95 (18.05) | 23.50 (6.72) | 23.85 (7.69) |
| Alanine Aminotransferase (U/L) | | 43.42 (49.90) | 26.20 (19.75) | 25.50 (10.15) |
| Alkaline Phosphatase (U/L) | | 84.11 (23.15) | 89.75 (30.81) | 96.35 (21.90) |
| Gamma Glutamyl Transferase (U/L) | | 34.84 (27.29) | 26.15 (18.31) | 35.80 (25.06) |
| Lactate Dehydrogenase (U/L) | | 173.42 (36.09) | 167.00 (22.43) | 161.60 (18.34) |
| Calcium (mg/dl) | | 9.40 (0.58) | 9.24 (0.32) | 9.36 (0.38) |
| Sodium (meq/L) | | 138.74 (1.97) | 139.60 (2.82) | 138.80 (2.19) |
| Potassium (meq/L) | | 4.91 (0.42) | 4.82 (0.62) | 4.68 (0.52) |
| Chlorine (meq/L) | | 97.76 (21.48) | 103.30 (3.31) | 102.55 (2.52) |
| Phosphorus (mg/dL) | | 3.57 (0.48) | 3.47 (0.43) | 3.47 (0.38) |
| Magnesium (mg/dL) | | 2.15 (0.20) | 2.18 (0.16) | 2.11 (0.14) |
| Leukocytes (miles/mL) | | 5.04 (1.18) | 5.09 (1.23) | 5.68 (1.78) |
| Hemoglobin (g/dl) | | 15.47 (1.75) | 15.49 (1.41) | 15.54 (1.77) |
| Platelets (miles/mL) | | 262.42 (60.83) | 254.95 (64.12) | 256.65 (64.99) |
| Neutrophils (miles/mL) | | 2.77 (0.93) | 2.81 (0.92) | 3.37 (1.33) |
| Lymphocytes (miles/mL) | | 1.63 (0.42) | 1.59 (0.40) | 1.65 (0.47) |
